# The interaction of silver(II) complexes with biological macromolecules and antioxidants

**DOI:** 10.1007/s10534-019-00198-0

**Published:** 2019-05-16

**Authors:** Katherine D. Trotter, Olawale Owojaiye, Stuart P. Meredith, Pat E. Keating, Mark D. Spicer, John Reglinski, Corinne M. Spickett

**Affiliations:** 1 Department of Pure & Applied Chemistry, Strathclyde University, 295 Cathedral Street, Glasgow, G1 1XL UK; 2 School of Life and Health Sciences, Aston University, Aston Triangle, Birmingham, B4 7ET UK; 3 Strathclyde Institute of Pharmacy and Biomedical Sciences, Strathclyde University, 161 Cathedral Street, Glasgow, G4 0NR UK

**Keywords:** Ag(II) 2,6-dicarboxypyridine, Antimicrobial metal, Glutathione, Lipid peroxidation, Oxidative stress

## Abstract

Silver is widely used for its antimicrobial properties, but microbial resistance to heavy metals is increasing. Silver(II) compounds are more oxidizing and therefore have the potential to overcome resistance via extensive attack on cellular components, but have traditionally been hard to stabilize for biological applications. Here, the high oxidation state cation was stabilised using pyridinecarboxylate ligands, of which the 2,6-dicarboxypyridine Ag(II) complex (Ag2,6P) was found to have the best tractability. This complex was found to be more stable in phosphate buffer than DMSO, allowing studies of its interaction with water soluble antioxidants and biological macromolecules, with the aim of demonstrating its potential to oxidize them, as well as determining the reaction products. Spectrophotometric analysis showed that Ag2,6P was rapidly reduced by the antioxidants glutathione, ascorbic acid and vitamin E; the unsaturated lipids arachidonic and linoleic acids, model carbohydrate β-cyclodextrin, and protein cytochrome c also reacted readily. Analysis of the reaction with glutathione by NMR and electrospray mass spectrometry confirmed that the glutathione was oxidized to the disulfide form. Mass spectrometry also clearly showed the addition of multiple oxygen atoms to the unsaturated fatty acids, suggesting a radical mechanism, and cross-linking of linoleic acid was observed. The seven hydroxyl groups of β-cyclodextrin were found to be completely oxidized to the corresponding carboxylates. Treatment of cytochrome c with Ag2,6P led to protein aggregation and fragmentation, and dose-dependent oxidative damage was demonstrated by oxyblotting. Thus Ag2,6P was found to be highly oxidizing to a wide variety of polar and nonpolar biological molecules.

**Electronic supplementary material:**

The online version of this article (10.1007/s10534-019-00198-0) contains supplementary material, which is available to authorized users.

## Introduction

The antiseptic and oligodynamic behaviour of silver has been appreciated for more than 200 years (Marx and Barillo 2014; Mijnendonckx et al. 2013). However, even in recent years its application as a biocide has been limited to the use of a few relatively simple preparations (Azócar et al. 2014). Many medical items such as catheters and prostheses are coated with the elemental form of silver, which prevents colonization of their surface by pathogens (Hetrick and Schoenfisch 2006). The release of silver cations through slow dissolution of silver halide is an effective method of disinfecting water for domestic consumption (Silvestry-Rodriguez et al. 2007). Since the late 1960s, silver sulfadiazine (SSD, or Flamazine®) has been common in anti-bacterial preparations for the treatment of burns (Atiyeh et al. 2007). Even this compound is a simple preparation comprising an antimicrobial cation and anion formulated together as a weakly bonded complex (Cook and Turner 1975). Although there has been robust interest in developing new silver-based anti-infective agents such as silver nanoparticles (Ag/Ag_2_O), novel silver complexes and silver impregnated fabrics (Ag^0^/Ag^+^) (Fromm 2013; Konop et al. 2016; Le Ouay and Stellacci 2015; Simoncic and Klemencic 2016; Singh et al. 2015), these are mainly reformulations of silver in its commonest Ag^0^/Ag^+^ forms.

The mechanisms of antimicrobial action of these silver formulations are still not completely understood, but the consensus is that they can act on a variety of targets, including interactions with bacterial cell membranes, binding to and inhibition of thiol-containing proteins, and release of reactive oxygen species through processes still to be fully elucidated (Konop et al. 2016). As Ag^+^ is a moderate oxidizing agent (Ag^+^ + e^−^ → Ag; E^0^ = 0.80 V), only the most sensitive redox sites are affected by it. Although it has sometimes been suggested that the multimodal mechanism of Ag^0^/Ag^+^ formulations make resistance less likely to develop, resistance to heavy metals in general is well known (Pal et al. 2014), and reports of resistance to silver compounds are on the increase (Hanczvikkel et al. 2018; Panacek et al. 2018; Percival et al. 2005). In view of the fact that antimicrobial resistance is a major problem worldwide and aggressive microbial species such as *E. coli* 0157, MRSA, and *C. difficile* are on the increase (Brandt et al. 2014; Heiman et al. 2015; Vindigni and Surawicz 2015), development of more powerful formulations of silver with higher oxidizing potential would be desirable, especially for external use in disinfection and cleansing.

In addition to Ag^0^ and Ag^+^, silver also has two higher oxidation states that are potentially extremely powerful oxidants: Ag^2+^ (Ag^2+^ + e^−^ → Ag^+^; E^0^ = 2.0 V) and Ag^3+^ (Ag^3+^ + e^−^ → Ag^2+^; E^o^ unknown) (Weast 1979). Biocidal silver compounds such as SSD are all based on compounds of silver in its less reactive, lower oxidation states (Ag^0^/Ag^+^). This choice, in part, was due to an inability to stabilize and control silver in its higher oxidation states in early synthetic studies. However, many of these issues have been resolved and routes are available for the production of a wide range of silver(II) compounds and a more limited range of silver(III) species (Levason and Spicer 1987). This opens the door for development of novel, high oxidation state silver compounds for antimicrobial disinfection.

Increasing the redox potential of the silver agent is an effective method of enhancing biocidal activity, as it limits the effectiveness of antioxidant defence. Powerful oxidants such as silver(II) can be expected to irreversibly chemically oxidize a wide range of functional (sulfhydryl, vicinal diols) and structural components (unsaturated lipid, proteins, carbohydrates) on the surface and inside the microbial cell. However, a change in oxidation state not only increases the redox potential, it also changes the preferred shape of the metal complex. Silver(I) has a marked preference for tetrahedral geometry, whereas d^9^ silver(II) predominantly adopts square planar geometry. It is known that the geometry a metal complex adopts can affect its biological activity; for example, the ability of platinum compounds to interact with DNA (Rosenberg et al. 1969) and the antimicrobial and anticancer activities of various metal complexes (Malik et al. 2018). Metal complexes can be transported across membranes by passive and active mechanisms (Martinho et al. 2018), and it has been reported that specific coordination structures may occur during active transport; for example, in N-MBD Cu^+^-ATPases Cu^+^ adopts a trigonal planar form (Arguello et al. 2012). Thus, biocides based on silver(II) could allow an enhanced oxidative attack and, depending on their geometry, might exert diverse effects on biological systems. There is a wide range of simple ligands which can be used to stabilize silver(II), but the pyridinecarboxylates are an excellent initial choice, as they have been synthesized previously and mostly form planar complexes (Drew et al. 1970; Drew et al. 1971; Fowles et al. 1968), although the silver(II) complex with 2,6-dicarboxypyridine has been reported to be octahedral (Drew et al. 1969). However, their ability to react with biological molecules and cause oxidative damage has not been studied previously. Consequently, silver(II) complexes were prepared using pyridinecarboxylates as ligands, and the stability of these complexes was investigated. The aim of the study was to determine the effectiveness of the silver(II) complexes for oxidizing biological antioxidants, lipids and proteins.

## Experimental

All reagents were obtained commercially. UV–Vis spectra were recorded on an Agilent Technologies Cary 60 UV–Vis spectrophotometer. NMR analysis was carried out on a Bruker AMX 400 operating at 400 MHz for ^1^H. Solid reflectance spectra (400–900 nm) were recorded on a Photonics CCD array UV–Vis spectrophotometer. Silver(II) complexes of 2-carboxypyridine, 2,3-dicarboxypyridine, 2,4-dicarboxypyridine, 2,5-dicarboxypyridine and 2,6-dicarboxypyridine (Ag2,6P) as were prepared using literature methods (Drew et al. 1970; Drew et al. 1971; Fowles et al. 1968).

### The protocol for handling Ag2,6P in solution

A reference sample of Ag2,6P was prepared using published methods (Fowles et al. 1968). The sample was subjected to elemental analysis (found: C 32.77, H 2.68, N 5.85%: expected for Ag2,6P·4H_2_O: C 32.97, H 2.77, N 5.49%), which confirmed the hydration state. This reference sample was used to calculate the molar extinction coefficient of Ag2,6P in water (ε_570_, 252/M/cm; ε_890_, 207/M/cm) and DMSO (ε_600_, 88.6/M/cm). Ag2,6P was found to decompose slowly with the natural green/black colour giving way to a white product. Hence small batches of Ag2,6P were prepared immediately prior to use to avoid problems associated with degradation, and given amounts of Ag2,6P were quickly dissolved in a given amount of solvent. The resulting solution was subjected immediately to spectrophotometric analysis and the concentration of Ag2,6P in solution calculated retrospectively using molar extinction coefficient obtained from the reference sample.

Thus although all the experiments were carried out in duplicate or triplicate, the difficulty of producing completely dry complex meant that it was impossible to generate solutions containing exactly the same concentration of reagents. Consequently, the data shown are derived from representative experiments.

### The stability of bis-(2,6-dicarboxypyridyl)silver(II) in solution

A solution of bis-(2,6-dicarboxypyridyl)silver(II) was prepared either in phosphate buffer (0.1 M KH_2_PO_4_, pH 7.0) at 4.47 mM or in DMSO at 18.4 mM. Aliquots of solutions were transferred immediately to a cuvette and the absorbance (400–1000 nm) was monitored over a 2 h period.

### Reaction of bis-(2,6-dicarboxypyridyl)silver(II) with glutathione

An 8.5 mM solution of Ag2,6P in phosphate buffer was prepared and 2.25 mL was transferred to a cuvette and the visible spectrum (400–900 nm) recorded. Aliquots (20 μL) of reduced glutathione (GSH) solution (84.3 mM) in phosphate buffer were added to the cuvette and the spectrum re-recorded after each addition until the band (λ_max_ 570 nm) attributed to silver(II) disappeared (~ 120 μL).

To investigate the products of the reaction using NMR, three solutions of reduced glutathione (6.1 mg in 1 mL of D_2_O, 20 mmol) were treated with 8.2 mg (18.5 mmol), 16.6 mg (36 mmol) or 24.4 mg (53 mmol) of Ag2,6P respectively. The solutions were allowed to react overnight and then filtered into a 5 mm NMR tube. ^1^H NMR spectra were obtained using a Bruker AVANCE 3 spectrometer operating at 400.12 MHz. Samples were maintained at 300 K during spectral acquisition. The NMR spectra were collected using a standard pulse sequence. The free induction decay was generated by a 3.13 μs pulse width corresponding to a 30^o^ pulse. Each data set (4 k scans; no water suppression) was collected in 32 k of memory. A 1 Hz line broadening function was applied before Fourier transformation to reduce the effect of the baseline noise.

### Reaction of bis-(2,6-dicarboxypyridyl)silver(II) with ascorbic acid

The reaction of bis-(2,6-dicarboxypyridyl)silver(II) (7.6 mM) with ascorbic acid (144 mM) was investigated essentially as described for glutathione. Aliquots (20 μL) of the ascorbate solution were added to a cuvette containing 2.25 mL of Ag2,6P and the spectrum re-recorded until the band (λ_max_ 570 nm) disappeared, which corresponded to the addition of 80 μL.

### Reaction of bis-(2,6-dicarboxypyridyl)silver(II) with vitamin E (α-tocopherol)

The visible spectrum (400–900 nm) of 2.25 mL of 9.1 mM bis-(2,6-dicarboxypyridyl)silver(II) in DMSO was recorded before and after addition of 10 μL aliquots of 54.3 mM α-tocopherol in DMSO, with loss of absorbance at 620 nm after addition of 70 μL. Due to the competition between the concurrent reactions of Ag2,6P with α-tocopherol and Ag2,6P with DMSO, a definite end point cannot be given for the reaction of Ag2,6P with α-tocopherol (vide infra).

### Reaction of bis-(2,6-dicarboxypyridyl)silver(II) with linoleic and arachidonic acid

A 2.25 mL aliquot of 19.5 mM bis-(2,6-dicarboxypyridyl)silver(II) in DMSO in a cuvette was reacted sequentially with 10 μL aliquots of 91.1 mM sodium linoleate or 98.7 mM sodium arachidonate, both prepared in DMSO. The reaction was monitored by recording the visible spectrum until the band (λ_max_ 620 nm) had disappeared. No definite end point could be given due to the competing reaction with DMSO occurring.

For analysis by mass spectrometry, 2 mL of aqueous suspensions of the sodium salts of the fatty acids (sodium linoleate; 16.5 mg, 54 μmoles or arachidonic acid; 16.5 mg, 50 μmoles) were reacted with 20 mg (45 μmoles), 40 mg (91 μmoles) or 60 mg (136 μmoles) of Ag2,6P. The solutions were allowed to react overnight and then filtered before analysis as described below.

### Reaction of bis-(2,6-dicarboxypyridyl)silver(II) with β-cyclodextrin

The reaction with β-cyclodextrin (25 mmol/L) with Ag2,6P was tested essentially as for ascorbic acid described above, except that the reaction was carried out in a sample bottle and 200 μL aliquots of β-cyclodextrin were added sequentially until the band at 570 nm had been extinguished (after addition of 2.0 mL).

For analysis by mass spectrometry, aqueous solutions (2 mL) of β-cyclodextrin containing 100 mg (88 μmoles) were treated with Ag2,6P (200 mg, 0.44 mmol). The solutions were allowed to react overnight and then filtered before analysis as described below.

### Electrospray mass spectrometric analysis of small molecules

Electrospray mass spectra were recorded using an Agilent 6130 (dual source). Samples of glutathione, vitamin E, linoleic acid, and arachidonic acid prepared as described above were diluted in methanol, while β-cyclodextrin was diluted in 50:50 acetonitrile containing 0.1% formic acid:water and introduced into the instrument with an infusion rate of 0.2 mL/min using methanol. Spectra were acquired with the following parameters: ionization mode, MM-ES + APCI, −ve ionization; source temperature, 300 °C; Voltage, 4000 V; Curtain gas flow rate, 12 L/min; m/z range 50–2000. Spectra were typically acquired for 30 s and averaged. The MS data were analysed using Agilent Chemstation.

### Reaction of bis-(2,6-dicarboxypyridyl)silver(II) with cytochrome-c

Aqueous solutions (2 mL) of cytochrome c (16.5 mg, 54 μmoles) were incubated with 20 mg (44 μmoles), 40 mg (88 μmoles) or 60 mg (131 μmoles) of Ag2,6P. The solutions were allowed to react overnight and then filtered before analysis as described below.

### Analysis of protein oxidation by oxyblotting for DNP-carbonyl adducts

Aliquots of the samples (10 µL; ~ 75 µg protein) were resolved by SDS-PAGE with a 12% resolving gel (Sambrook and Russell 2006) and then either stained with InstantBlue stain (Sigma-Aldrich, UK) or transferred onto PVDF membrane for oxyblotting as described previously (Shacter 2000). After washing, the membrane was acidified with 2 N HCl and labelled with 10 mM dinitrophenylhydrazine (DNPH) (Sigma-Aldrich, UK) for 5 min. After further washing and blocking the membrane was incubated in blocking buffer containing monoclonal primary antibodies rabbit anti-dinitrophenylhydrazone (anti-DNP) (D9656, Sigma-Aldrich, UK) at a working dilution of 1:1000 overnight at 4 °C. The secondary antibody was HRP-linked goat anti-mouse (6154, Sigma-Aldrich, UK) antibody (working dilution 1:1000) for 2 h at room temperature. The membrane was washed again as described above and HRP-linked anti-mouse was detected using enhanced chemiluminescence (ECL kit 34078, Thermo Fisher Scientific, Hemel Hempstead, UK) according to the manufacturer’s instructions. The membrane was scanned using a G:BOX system (Syngene, Cambridge, UK) running the GeneSys software (Syngene, Cambridge, UK).

## Results and discussion

### Solubility and stability of silver(II) complexes

**Fig. 1:**
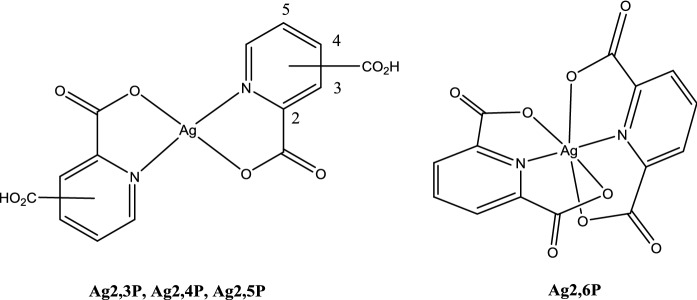
Structures of the silver (II) complexes synthesized. 2,3-dicarboxypyridine (Ag2,3P), 2,4-dicarboxypyridine (Ag2,4P), 2,5-dicarboxypyridine (Ag2,5P) and 2,6-dicarboxypyridine (Ag2,6P)

Silver(II) complexes of 2-carboxypyridine (Ag2P), 2,3-dicarboxypyridine (Ag2,3P), 2,4-dicarboxypyridine (Ag2,4P), 2,5-dicarboxypyridine (Ag2,5P) and 2,6-dicarboxypyridine (Ag2,6P) were prepared using literature methods (Drew et al. 1970; Drew et al. 1971; Fowles et al. 1968). Ag2P, Ag2,3P, Ag2,4P and Ag2,5P had limited solubility in both water and DMSO, and were not studied further owing to the limited relevance to biological environments. In contrast, Ag2,6P was observed to be reasonably soluble in both water (~ 10 mM) and DMSO (~ 20 mM). The solubility profile of the compounds most likely arises from their solid state structures: Ag2P and Ag2,3P are planar species and prone to π-stacking in the solid state (Fowles et al. 1968), which is known to affect solubility detrimentally. In contrast, Ag2,6P adopts an octahedral geometry in the solid state and is unable to π-stack, which lowers the lattice energy and promotes its solubility in polar solvents (Drew et al. 1970). The structures are shown in Fig. 1. Thus, despite the desirability of planar compounds for cell membrane transport, in the case of Ag(II) their potential may be limited by their lack of solubility.

In view of the high redox potential of silver(II) (equation 2; E^0^ = 2.0 V NHE (Weast 1979)), the silver (II) compounds were expected to be quite reactive even to the extent of potentially oxidizing water. Therefore, the first step was to investigate the lifetime of Ag2,6P in aqueous buffer and DMSO, by monitoring the visible absorbance spectrum (Suppl. Fig. 1a). In aqueous solution a small but manageable degradation (~ 10%) of Ag2,6P was observed over a 2 h period. The profile of the degradation process was linear within the lifetime of the experiment, suggesting that decomposition does not occur via an SN_2_ displacement of the axial carboxylates by water or phosphate. In contrast, the stability of Ag2,6P in DMSO was poor, having a half life of only 25 min (Suppl. Fig. 1b). DMSO can be oxidized to dimethylsulphone (Me_2_SO_2_; E^0^ = 1.54 V vs. NHE) and the silver(II) complex studied here is therefore theoretically capable of driving this reaction (Krtil et al. 1996). Solid reflectance spectrophotometry indicated that the solid and DMSO solution phase structures of Ag2,6P are similar (λ_max_ 600 nm), suggesting that decomposition occurs via electron transfer rather than ligand exchange. The reaction of the silver complex with DMSO limits the interpretation of reactions with other compounds carried out in this solvent, but in some cases there was no feasible alternative. To obviate problems with the slow decomposition of Ag2,6P in solution, fresh solutions were prepared immediately before the start of each experiment.

### Reaction with antioxidants

**Fig. 2:**
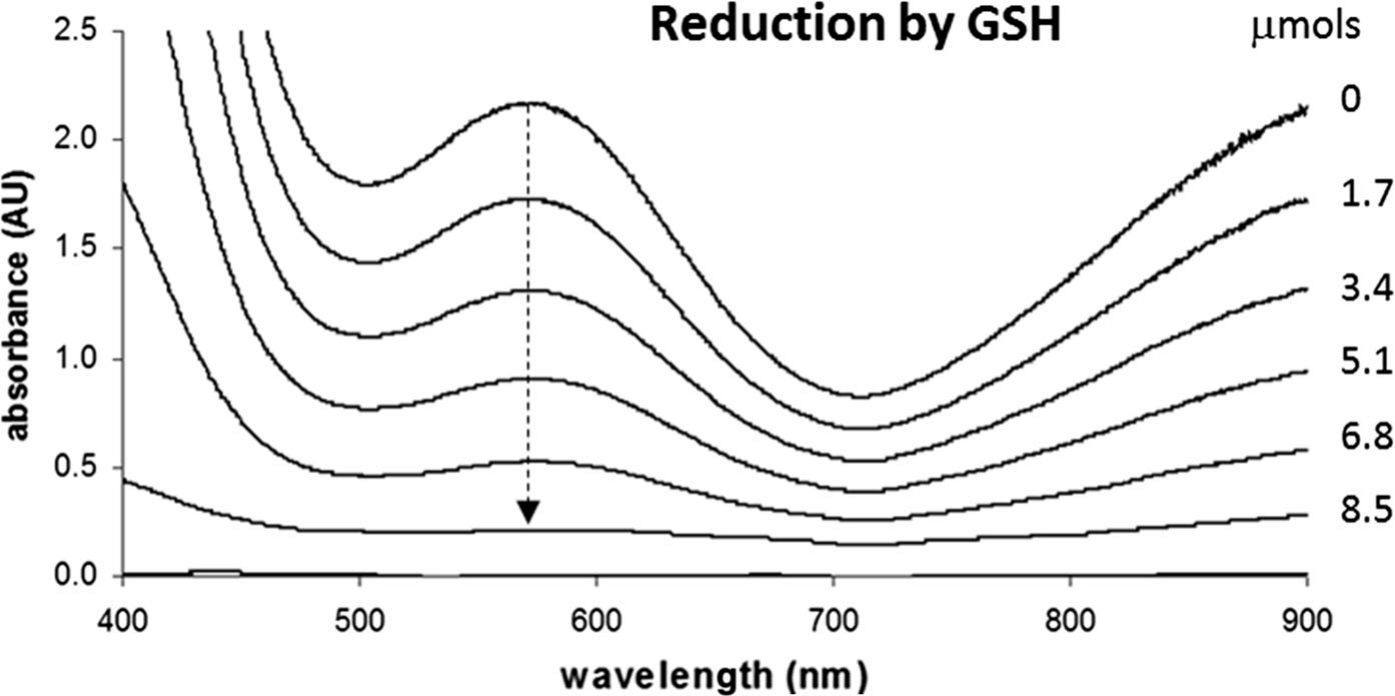
Spectrophotometric analysis of the reaction between Ag2,6P and glutathione. The titration of 8.5 mM Ag2,6P (18.7 μmoles in 2.2 mL) in 0.1 M KH_2_PO_4_, pH 7.0 with glutathione (GSH). The glutathione (84.3 mM) was added in 20 uL aliquots (a total of 6) and the corresponding μmoles of GSH are indicated on the right-hand side of the traces

Biological systems utilize a number of species as co-factors and reducing agents (e.g. glutathione, ascorbic acid, α-tocopherol), and depletion or oxidation of antioxidants and structural biological molecules is an early stage in the stress leading to the toxic effects of oxidizing compounds (Halliwell and Gutteridge 1998). Consequently, the reactions of Ag2,6P with these three antioxidants was investigated. The reaction with an antioxidant can readily be inferred by spectrophotometric titrations in which the stepwise reduction of the coloured Ag2,6P to its colourless silver(I) product is observed. The reactions of glutathione and ascorbate were carried out in aqueous solution, whereas the reaction of α-tocopherol was carried out in DMSO. Using the diminution of the band at 570 nm it was possible to titrate Ag2,6P with glutathione (Fig. 2), and the molar ratio at the end point was calculated to be slightly greater than 2:1 Ag2,6P:GSH. This would be generally consistent with the 2-electron oxidation of GSH to GSSG, assuming that the Ag2,6P undergoes a 1-electron reduction to Ag^+^, as the appearance of metallic silver was not observed.

**Fig. 3:**
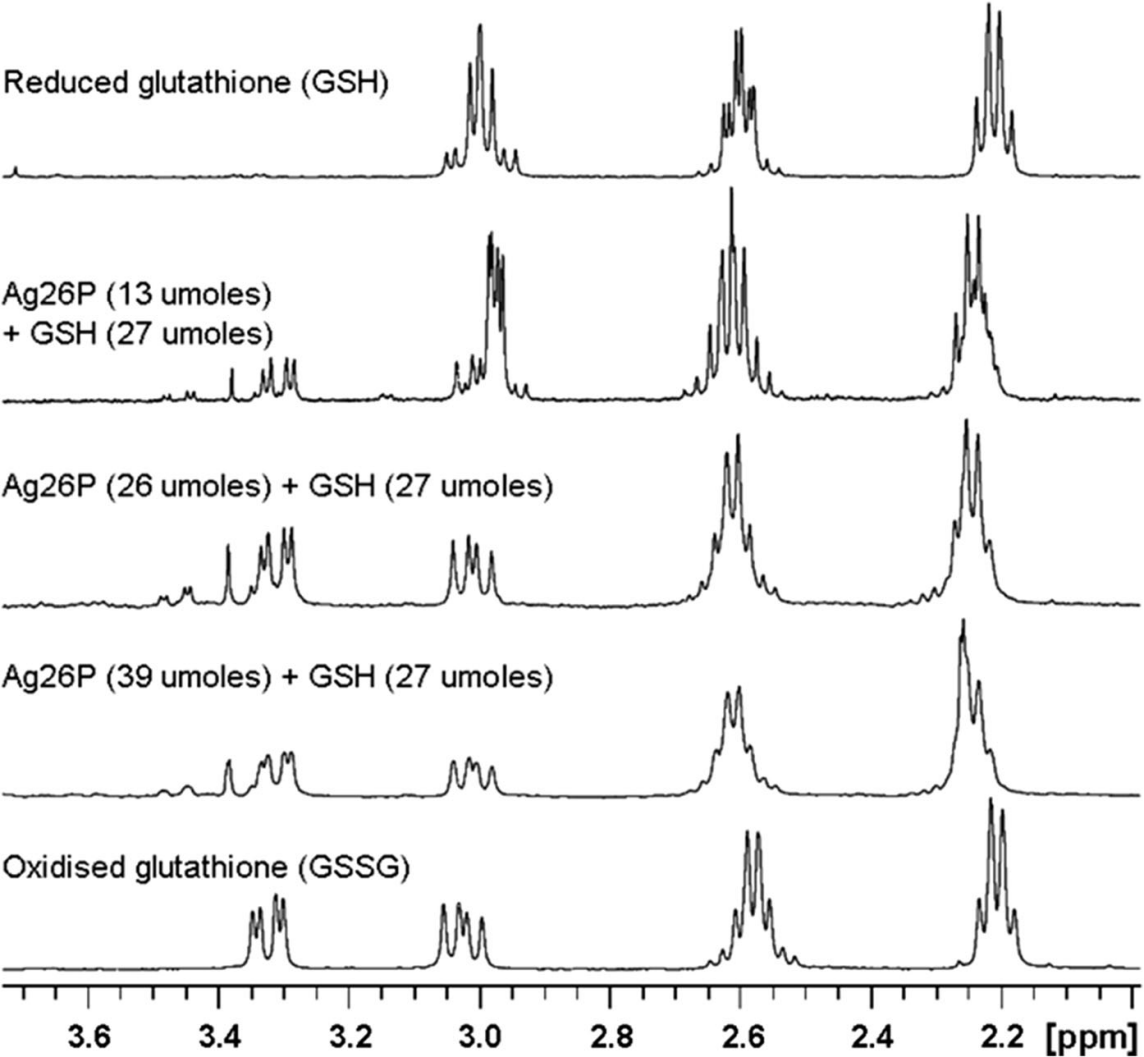
^1^H-NMR analysis of the reaction between Ag2,6P and glutathione. 27 µmols of glutathione (GSH) was reacted with increasing amounts of Ag2,6P as indicated on the spectra. The spectrum of commercial glutathione disulfide is shown at the bottom for comparison

To investigate the nature of the oxidation in more depth, the reaction of GSH was monitored using ^1^H-NMR. Figure 3 clearly demonstrates that Ag2,6P oxidized GSH to the disulfide form (GSSG), with increasing Ag2,6P amounts correlating with increased loss of the GSH triplet signals at ~ 2.95 ppm and appearance of the GSSG pairs of doublets at ~ 3.05 and 3.3 ppm. This finding was supported by negative ion electrospray mass spectrometry analysis (Suppl Fig. 2), which clearly showed that treatment of GSH (m/z 306.1) with Ag2,6P resulted in conversion to GSSG (m/z 611.1).

The reaction of Ag2,6P with ascorbic acid was monitored spectrophotometrically in the same way as that of glutathione, and it could clearly be seen that addition of ascorbic acid resulted in loss of absorbance at 570 nm (Suppl. Fig. 3). The molar ratio at the end point was calculated to be approx. 2:1 Ag2,6P:ascorbate, consistent with the 2-electron oxidation of ascorbate to dehydroascorbate. However, this reaction was not investigated further due to the lack of stability of dehydroascorbate in the presence of redox metals; metal-mediated ascorbic acid oxidation and redox cycling is a facile process involving low as well as high valent metals (Halliwell and Gutteridge 1998; Skov and Vonderschmitt 1975).

The reaction of α-tocopherol with Ag2,6P in DMSO was also discernable using spectrophotometry (Suppl. Fig. 4). However, due to the competing interaction of Ag2,6P with DMSO discussed above, it was not possible to obtain an accurate end point for the titration.

### ESI–MS analysis of the reaction with fatty acids

The unsaturated fatty acids linoleic acid and arachidonic acid were used as models to investigate the ability of Ag2,6P to oxidize lipid. Preliminary studies by spectrophotometric analysis indicated that reactions occurred, but the reactions were so fast that kinetic analysis was not possible; moreover the experiments were carried out in DMSO as the fatty acid salts were sparingly soluble in aqueous solution, and therefore were limited by the issues with this solvent mentioned above. The focus of these studies was therefore the analysis of oxidation products of the biomolecules.

**Fig. 4:**
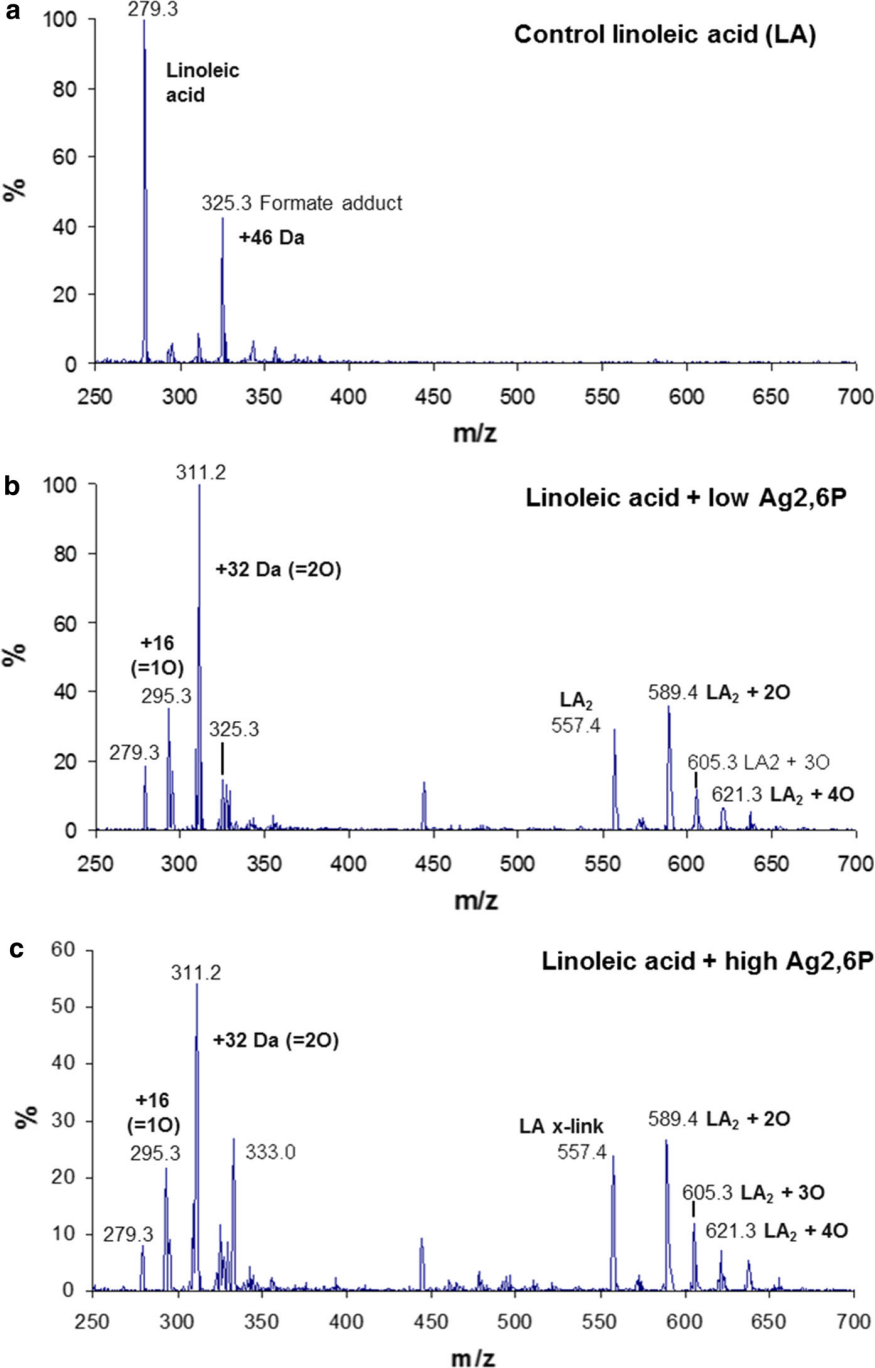
The reaction of linoleic acid with Ag2,6P studied by ESI-mass spectrometry in negative ion mode. **a** Untreated linoleic acid. **b** 29 mM Linoleic acid treated with 37 mM Ag2,6P and **c** 29 mM linoleic acid treated with 110 mM Ag2,6P

ESI-mass spectrometry was used to monitor the reaction of Ag2,6P with the unsaturated fatty acid linoleic acid (Fig. 4). A small amount of adventitious oxidation of the control sample is apparent in Fig. 4a, but the major signal is the native fatty acid at m/z 279 ([M–H]^−^). There was also a strong signal at m/z 325, which was identified as the formate adduct of linoleic acid ([M + CHO_2_]^−^). At the lower treatment concentration (Fig. 4b) the signal of the native lipid was greatly reduced and the major signal was at m/z 311, corresponding to the addition of O_2_ (+ 32 Da). There was also evidence of addition of a single oxygen atom at m/z 295, and another signal at m/z 293, which was most probably due to loss of water from the species at m/z 311, suggesting that it may be a bis-hydroxide rather than a hydroperoxide (Spickett and Pitt 2015). Interestingly, a peak was observed at m/z 557, which was consistent with the formation of a cross-linked dimer of linoleic acid (loss of 3H but singly charged) and there were also dimers containing 2, 3 and 4 additional oxygens. Crosslinking of oxidized fatty acyl chains under highly oxidizing conditions has been reported previously (Muizebelt and Nielen 1996; Schroter et al. 2016; Tolvanen et al. 2008). At the higher treatment concentration the native lipid was further depleted, probably resulting from degradation of the lipid to small, non-ionized breakdown products, but otherwise the oxidation pattern was comparable.

**Fig. 5:**
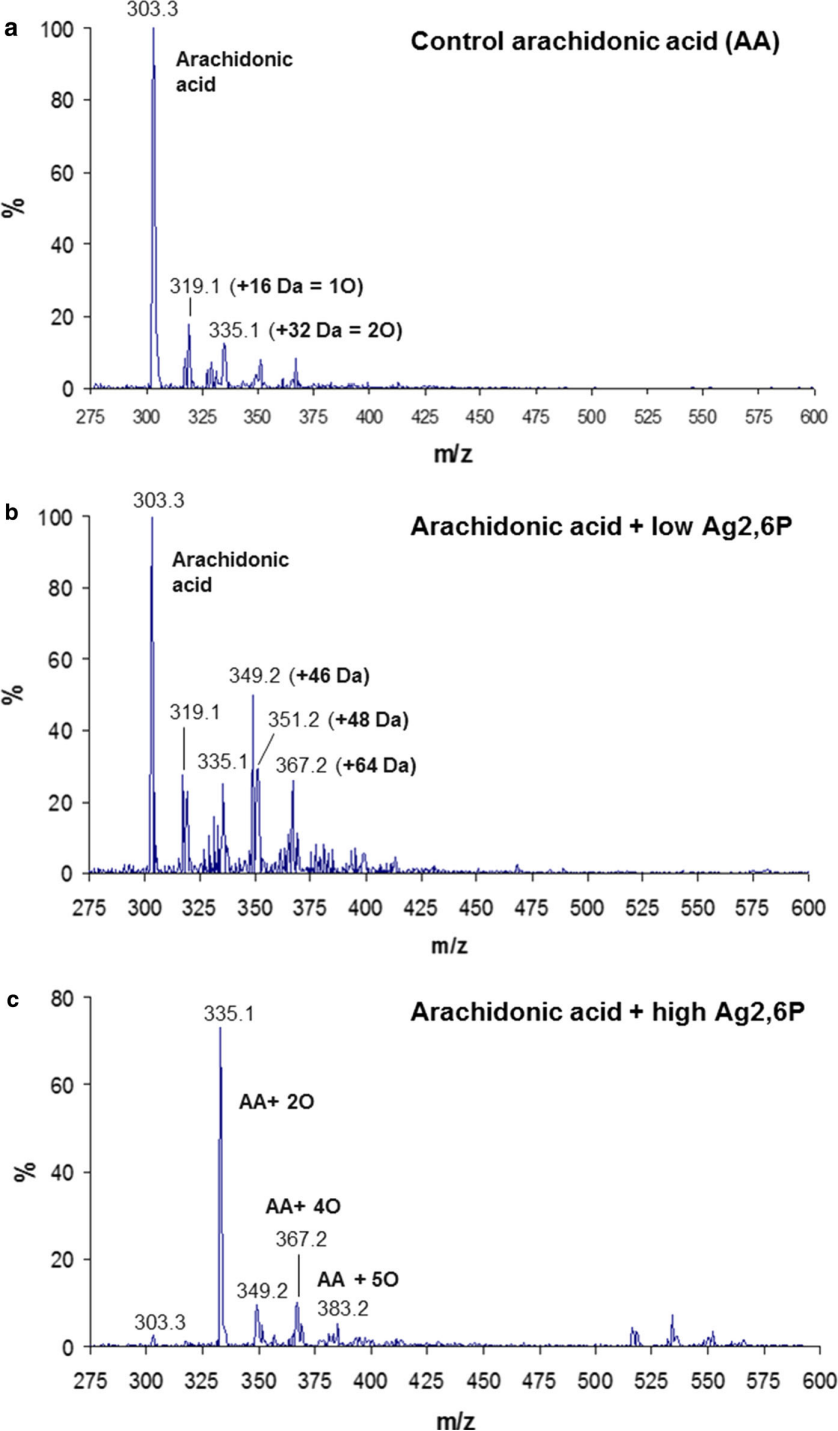
The reaction of arachidonic acid with Ag2,6P studied by negative ion ESI-mass spectrometry. **a** ESI-mass spectra of untreated arachidonic acid. **b** arachidonic acid (27 mM) treated with Ag26P (22.5 mM) and **c** arachidonic acid (27 mM) treated with Ag26P (68 mM). All samples were diluted equivalently in methanol prior to infusion into the instrument. The signal at m/z 349 is probably a formate adduct, while the one at 333 appears to be a contaminant

Treatment of arachidonic acid with Ag2,6P also clearly showed the occurrence of oxidation (Fig. 5). As with linoleic acid, there was some adventitious oxidation in the untreated sample, reflecting the susceptibility of this polyunsaturated fatty acid to autoxidation (Fig. 5a), but even at the low treatment concentration (Fig. 5b) the incorporation of oxygen was increased, with signals at m/z 319 (1 O), m/z 335 (2 O), m/z 351 (3 O) and m/z 367 (4 O). A strong signal at 349.2 was also observed, corresponding to + 46 Da; in view of its appearance only in treated samples, this is likely to be a dehydration product following the addition of 4 oxygens such as an epoxyisoprostane, which are known as relatively stable products of arachidonic acid (Spickett and Pitt 2015). At the higher treatment concentration the native signal at m/z/303 was almost abolished (Fig. 5c), and the fatty acid was more highly oxidized with the strongest signal at m/z 335.1 and all other products at higher masses and levels of oxidation. In contrast, there was no evidence of dimers of arachidonic acid analogous to those observed with linoleic acid, which should have occurred at m/z 605 (data not shown), although the ions that appeared between m/z 515–553 were not identified. Comparing the reactions of linoleic and arachidonic acids with Ag2,6P, it was clear that higher concentrations of the silver were required to deplete the more unsaturated fatty acid, reflecting its greater capacity for oxidative modification.

### Reaction with the carbohydrate beta-cyclodextrin

**Fig. 6:**
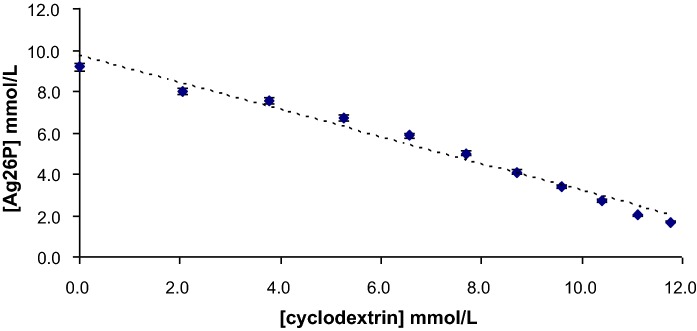
Spectrophotometric analysis of the reaction of β-cyclodextrin with Ag2,6P. 7.6 mM Ag2,6P (17.2 µmol in 2.2 mL) in 0.1 M KH2PO4, pH 7.0 was titrated against β-cyclodextrin (β-CD). The β-cyclodextrin (25.6 mM) was added in 200 µL aliquots (× 10). The mole ratio at the end point was calculated to be 1:3 Ag2,6P: β-CD mol:mol

**Fig. 7:**
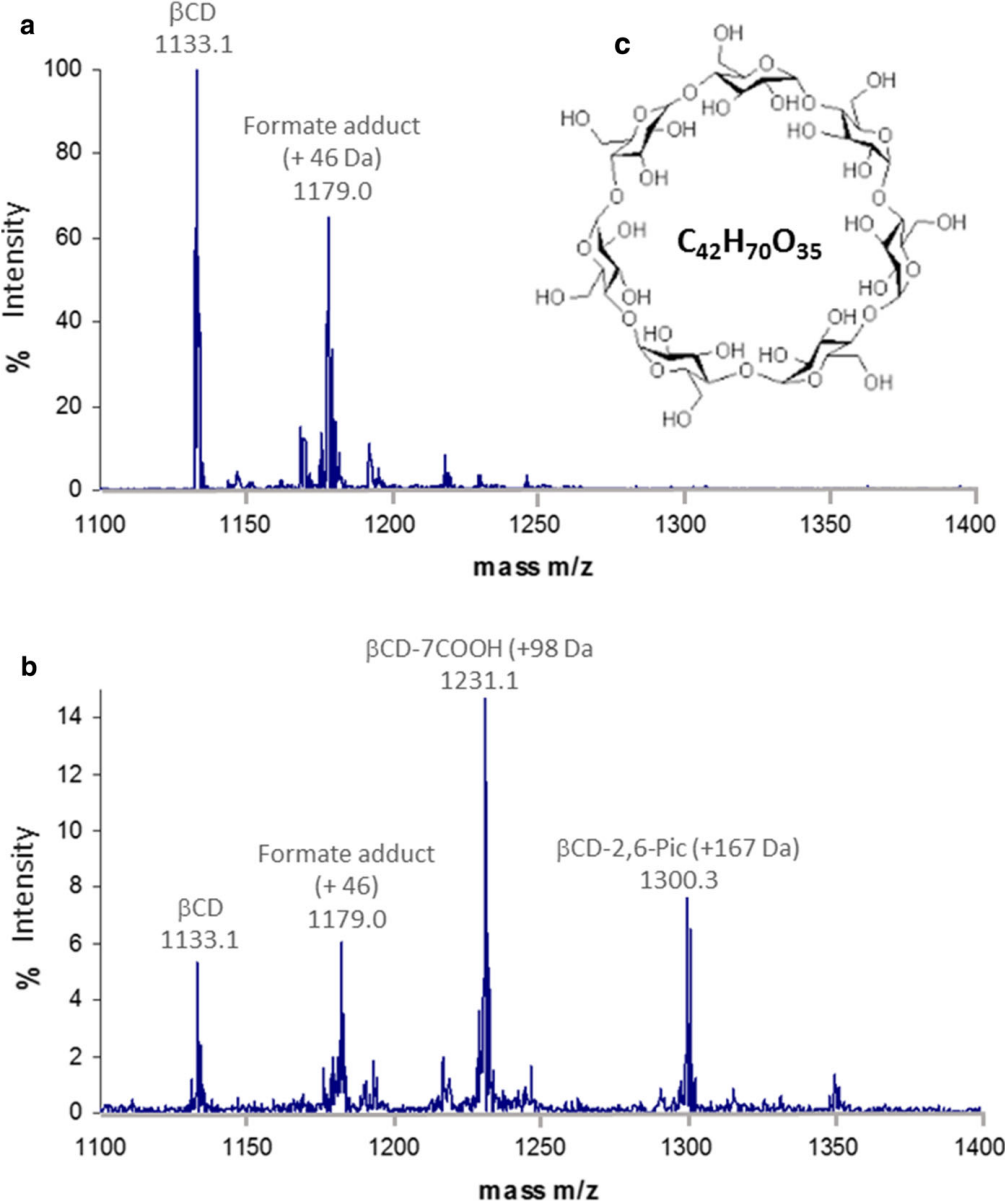
Electrospray mass spectrometry analysis of the reaction of Ag2,6P with β-cyclodextrin **a** the negative ion ESI-mass spectra of β-cyclodextrin, also showing a formate adduct at + 46 Da; **b** β-cyclodextrin (100 mg, 88 μmoles) treated with a 5-fold excess of Ag2,6P (200 mg, 0.44 mmol); and **c** structure of β-cyclodextrin together with its formula. Note that the y-axis scale is relative signal intensity, where in **a** the native β-cyclodextrin is the major species and therefore 100%, whereas in **b** 2,6-picolinate at m/z 166.1 was the major species at 100%

To investigate the effects of Ag2,6P on carbohydrates, β-cyclodextrin was used as a model, as it can readily be observed by mass spectrometry ([M-H]^−^ at m/z 1133), and moreover its oxidation by HOCl has been studied previously (Fraschini and Vignon 2000). Spectrophotometric titrations in aqueous solution (400–900 nm) clearly showed evidence of a reaction of Ag2,6P with β-cyclodextrin (Fig. 6). An end point was obtained at approximately 3:1 Ag2,6P: β-CD, which suggests that multiple oxidations might be occurring. Analysis by mass spectrometry also suggested that oxidation had occurred (Fig. 7). Figure 7a shows untreated β-cyclodextrin, which was the major species in the sample at m/z 1133.1 and therefore 100% relative intensity. As the samples were prepared in solvent containing formate, there was also a significant formate adduct at 1179.0. Treatment of the β-cyclodextrin with a 5-fold excess of Ag2,6-P led to free 2,6-picolinate presenting the strongest signal (100%) at m/z 166.1 (data not shown), while the β-cyclodextrin signal was substantially depleted and a signal at m/z 1231.1 appeared, corresponding to the oxidation of all of the hydroxyl groups into carboxylic acid to form a hepta-carboxylate β-cyclodextrin (plus 7 × 14 Da). A 2,6-dicarboxypyridine adduct of cyclodextrin at m/z 1300.3 was also observed, probably reflecting the high level of the free ligand present in the sample after reduction of Ag2,6-P by the carbohydrate. In contrast to the treatment of β-cyclodextrin with HOCl reported previously [20], there was no clear evidence of intermediate oxidation products, such as tris and hexakis carboxylate species, presumably owing to the highly oxidizing nature of the Ag^2+^ complex.

### Reaction with cytochrome c

**Fig. 8:**
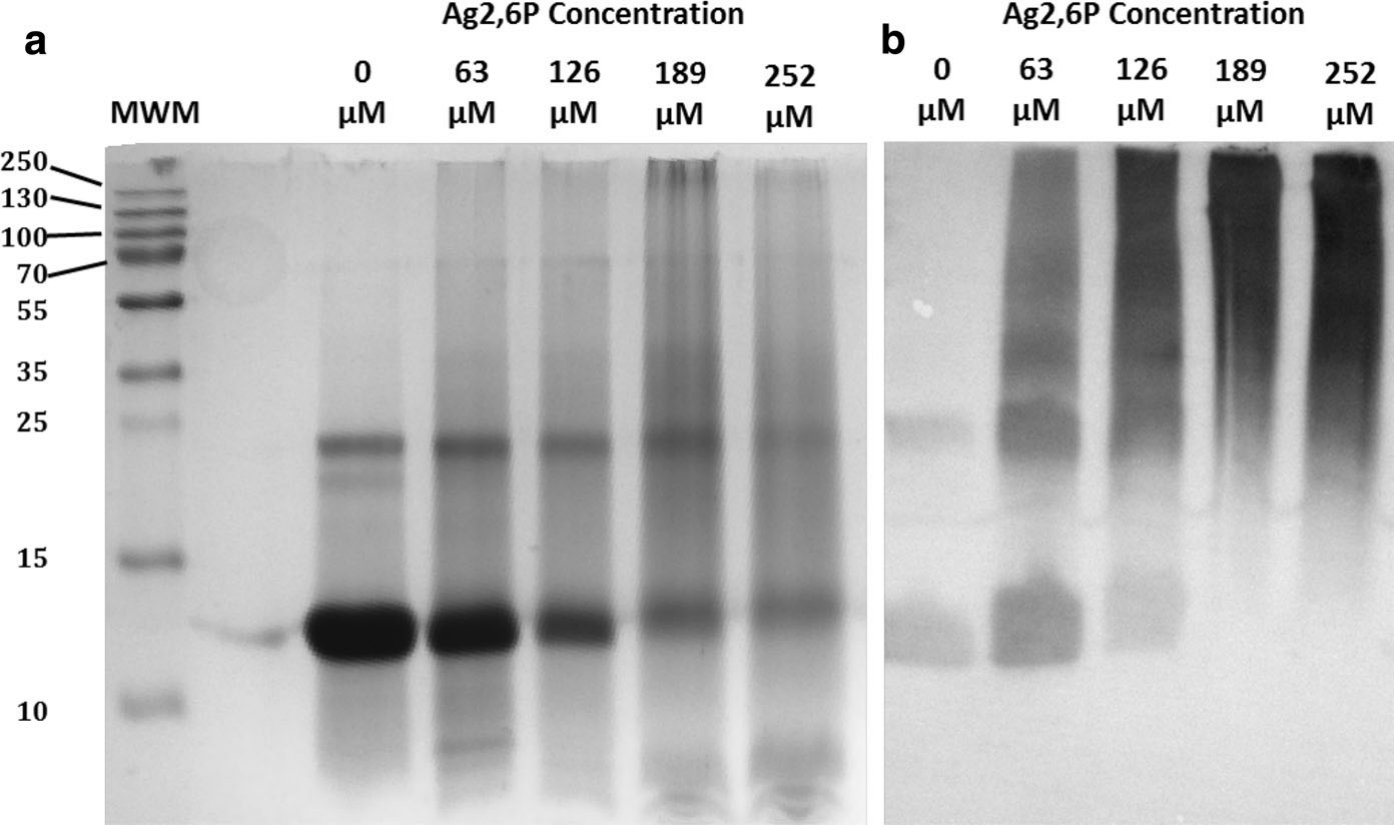
The effect of Ag2,6P treatment on Cytochrome c. Protein (8.25 mg/mL) was treated with 0, 63, 126, 189 or 252 µM Ag2,6P overnight. Molecular weights markers (MWM) are shown on the lefthandside with numbers in kDa; the molecular weight of horse heart Cytc is 12,384 Da. **a** Coomassie blue-stained 12% SDS-PAGE reducing gel. **b** Oxyblot of a comparable gel using an anti-DNPH primary antibody to determine the formation of carbonyl groups on cytochrome c as a marker of oxidative damage

Many adverse effects of oxidizing antimicrobial agents are mediated by protein oxidation and disruption of cell signaling, so the effect of Ag2,6P on cytochrome C as a model ubiquitous protein was studied. Preliminary studies by spectrophotometry showed that Ag2,6P was consumed by relatively small amounts of protein (data not shown), which is consistent with the presence of multiple oxidation sites on the polypeptides. To confirm the oxidative action of Ag2,6P on the protein, the formation of protein carbonyls was investigated, as these are well-established oxidation products (Domingues et al. 2013; Shacter 2000). Carbonyl formation may occur by oxidative deamination of lysines, or radical attack and fragmentation of various other residues (Davies 2016). The samples were first separated on denaturing polyacrylamide gels and visualized by staining with coomassie blue; then labeling of carbonyl groups with DNPH followed by western blotting with antibody to the DNP-adduct (commonly known as oxy-blotting) was carried out. Figure 8 shows that even low levels of Ag2,6P resulted in loss of the cytochrome c band at 12.3 kDa and appearance of high molecular weight aggregates that were retained at the top of the resolving gel, while higher concentrations led to more aggregates and additionally some degradation products observed at the bottom of the gel (Fig. 8a). Oxyblotting for oxidative damage to the protein showed the presence of increased carbonyls with increasing severity of the Ag2,6P treatment, initially in the cytochrome c band but this was lost at higher Ag2,6P concentrations and staining of the high molecular weight aggregates predominated (Fig. 8b). This clearly confirmed that extensive protein oxidation occurred following Ag2,6P treatment of the cytochrome c.

## Concluding remarks

Although planar metal complexes have been reported to be useful in terms of their ability to penetrate cell membranes, it is clear that for Ag(II) dicarboxypyridine complexes, the planar ones have lower aqueous solubility compared to one with octahedral geometry, namely Ag(II) 2,6-dicarboxypyridine (Ag2,6P). This compound has satisfactory stability in aqueous solution and was found to oxidize a variety of biomolecules. It was shown to react readily with the thiol-containing antioxidant glutathione, as well as with ascorbic acid (vitamin C) and a-tocopherol (vitamin E). It caused extensive oxidation of polyunsaturated fatty acids as well as cross-linking of the chains, and induced carbonyl formation, a common marker of oxidative damage, on the small model protein cytochrome c. Together, these effects suggest a one-electron or free radical mechanism of action, although it is not clear whether it involves direct reaction of the metal ion with the biomolecules, or intermediate action via oxidation of the solvents H_2_O or DMSO, leading to production of hydroxyl radicals (HO^·^) or other radical species.

The reactivity of Ag2,6P with a range of different biomolecules suggests that it is likely to have significant toxicity to bacterial cells and therefore good biocidal potential. Its solubility in the organic solvent DMSO implies that it may be able to penetrate lipid membranes and thus gain access to the intracellular environment, as lipophilicity is known to be a factor in biological transport of metal complexes (Oldfield et al. 2007). The reaction with beta-cyclodextrin is important and similar reactions could contribute to microbial toxicity by altering carbohydrates in the cell wall or on the surface of microbial cells.

However, Ag(II) complexes remain challenging to work with, owing to their limited stability in relevant solvents or even in pure form at room temperature. Although the study has shown that it should be possible to design improved biocides based on silver in a +2 formal oxidation state, further effort is required to design new ligands that support and target this moiety better.

## Electronic supplementary material

Below is the link to the electronic supplementary material.

Supplementary material 1 (PDF 283 kb)

## Data Availability

The original data associated with this project are available at 10.17036/researchdata.aston.ac.uk.00000418.
