# Supplementary material for: The interaction of silver(II) complexes with biological macromolecules and antioxidants
Source: Biometals. 2019 May 16;32(4):627–40. doi: 10.1007/s10534-019-00198-0 (PMC6647504; doi:10.1007/s10534-019-00198-0)
Supplement: Supplementary file 1 — Supplementary material 1 (PDF 283 kb) [file 10534_2019_198_MOESM1_ESM.pdf]

Journal: Biometals Date: 2019

The interaction of silver(II) complexes with biological macromolecules and antioxidants

Katherine D. Trotter, Olawale Owojaiye, Stuart Meredith, Pat E. Keating, Mark D. Spicer, Corinne M. Spickett,and John Reglinski

Corresponding author: Prof Corinne M. Spickett; Tel: 0121 2044085; E-mail: c.m.spickett@aston.ac.uk; Orcid: https://orcid.org/0000-0003-4054-9279

Supplementary Figures

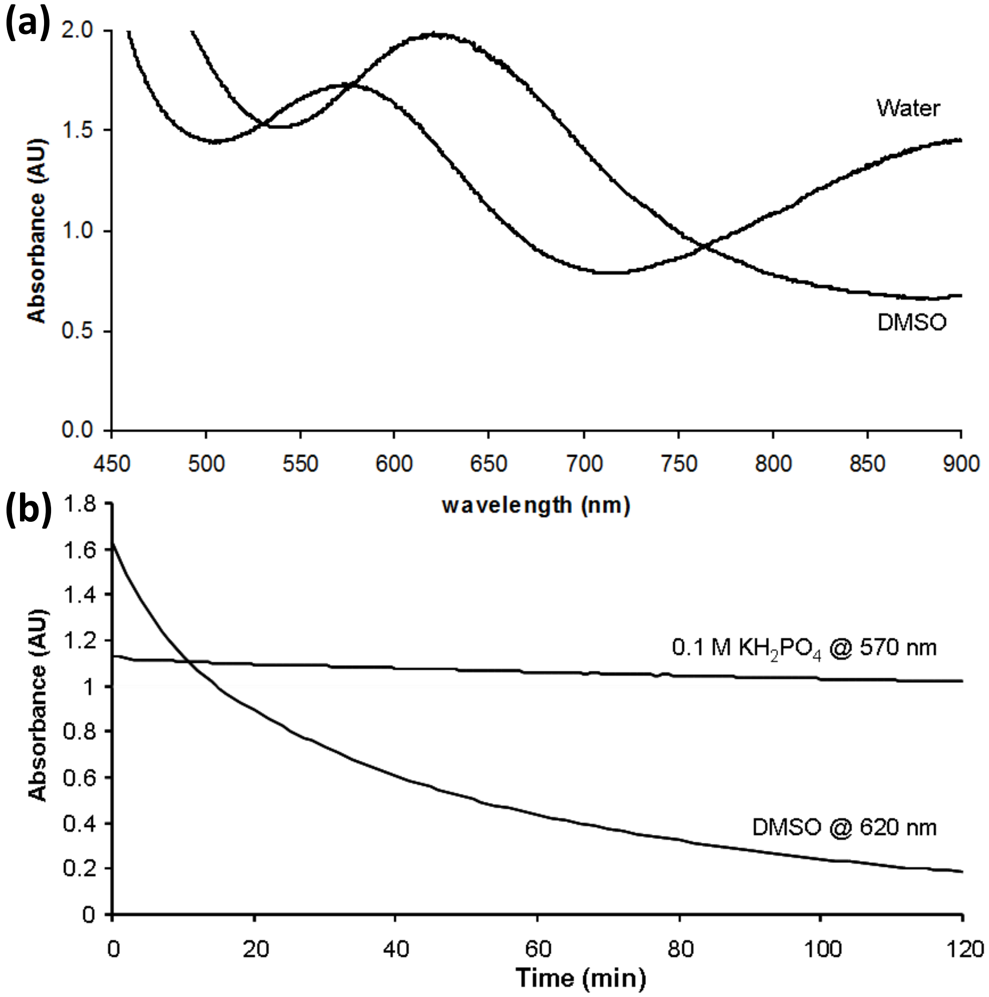

Supplementary Fig 1 Spectra and stability of Ag26P in phosphate buffer and DMSO. (a) Typical visible spectra of Ag26P in water (8.84 mM) and DMSO (21.5 mM), showing that the λmax was shifted in DMSO compared to water. (b) Timecourses of decomposition of Ag26P were monitored via the diminution of the bands at 590 nm (water) or 620 nm (DMSO) over a 2 hr period.

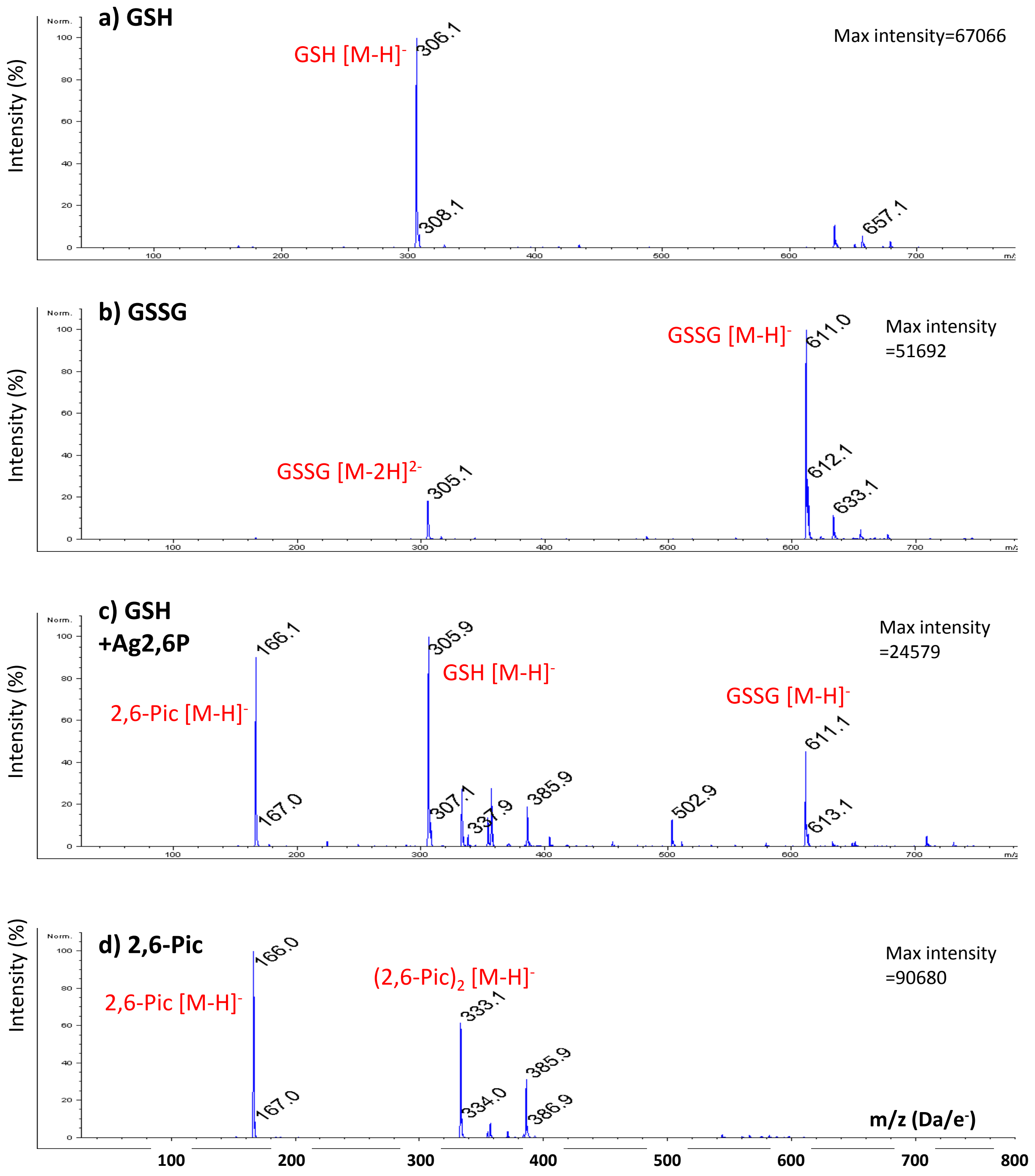

Supplementary Figure 2. Negative ion ESI-MS of GSH and GSSG treated with Ag2,6P. A) 20 mM GSH solution; B) 10 mM GSSG solution; C) 20 mM GSH treated with approx. equimolar Ag2,6P and incubated overnight; D) the ligand 2,6-dicarboxypyridine at ~20 mM for comparison and to show the source of signals appearing in the treated glutathione samples.

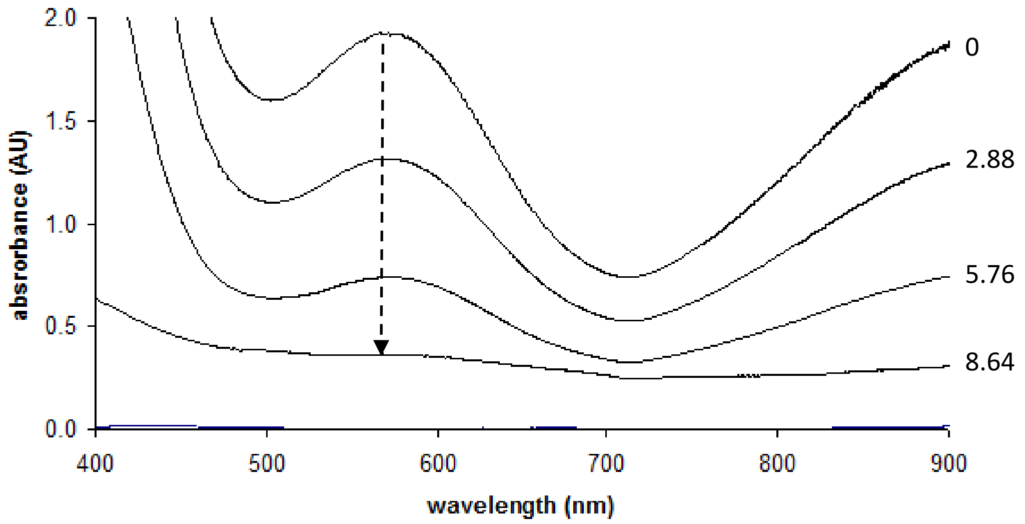

Supplementary Figure 3. The titration of 19 μmoles Ag26P (7.6 mM in 0.1 M KH_2PO_4, pH 7.0) with ascorbic acid. The ascorbic acid (144 mM) was added in 20 μL aliquots and the amounts are shown on the right hand side of the spectra.

(a)

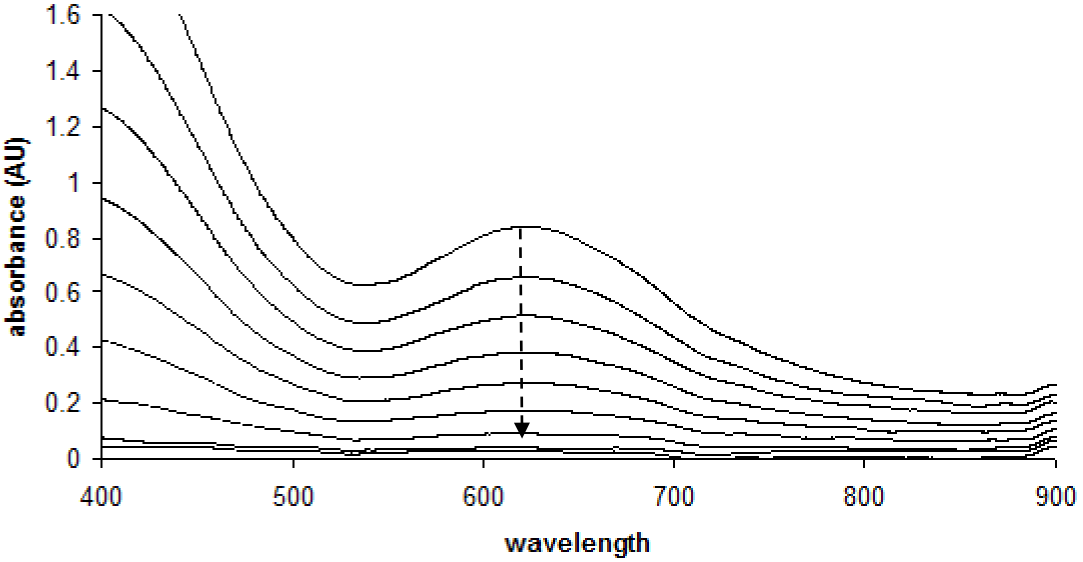

Supplementary Figure 4. The titration of 9.11 mM Ag26P in DMSO with α-tocopherol. The α-tocopherol (54.3 mM) was added in 20 μL aliquots. The mole ratio at the nominal end point cannot be calculated due to the competing degradation process shown in Suppl. Fig 1.
